# Association of life’s essential 8 with pulmonary function impairment: a cross-sectional analysis of the Kailuan study

**DOI:** 10.3389/fnut.2026.1750094

**Published:** 2026-05-28

**Authors:** Yanhui Deng, Aitian Wang, Yujie Ma, Jingtao Liang, Hualing Zhao, Lulu Chu, Jingli Gao

**Affiliations:** 1 Graduate School of Hebei North University, Zhangjiakou, China; 2 Department of Intensive Medicine, Kailuan General Hospital, Tangshan, China; 3 Department of Endocrinology, Kailuan General Hospital, Tangshan, China

**Keywords:** cardiovascular health, cross-sectional study, Kailuan study, Life’s Essential 8, lung function, pulmonary function impairment

## Abstract

**Background:**

Pulmonary function impairment not only reflects underlying respiratory disease but also serves as an independent predictor of systemic morbidity, particularly cardiovascular disease. Life’s Essential 8 (LE8), a composite cardiovascular health (CVH) metric endorsed by the American Heart Association, encompasses four health behaviors and four health factors. Despite its comprehensive nature, direct evidence on the relationship between LE8 and pulmonary function impairment remains scarce.

**Methods:**

Between 2015 and 2023, a total of 17,191 adults (with 97.8% men and 2.2% women) in the Kailuan study underwent pulmonary function testing, pulmonary function impairment was defined as percent-predicted FEV₁ < 80% or percent-predicted FVC < 80%. LE8 scores were calculated using American Heart Association algorithms and categorized as low (0–49), moderate (50–79), or high (80–100). Logistic regression were used to estimate odds ratios (ORs) and 95% confidence intervals (CIs) for impairment across LE8 categories and per 10-point increment. Dose–response associations were examined with restricted cubic splines, population attributable risk percent (PAR%) was estimated, component importance was evaluated using weighted quantile sum (WQS) regression, and robustness was assessed through subgroup and sensitivity analyses.

**Results:**

Mean age was 43.6 years and pulmonary function impairment prevalence was 33.4%. The mean LE8 score was 60.2 (low *n* = 3,395; moderate *n* = 13,069; high *n* = 727). Impairment prevalence was 38.6, 32.3, and 28.2% across low, moderate, and high groups (p for trend <0.001). In adjusted logistic regression models, ORs (vs. low) were 0.80 (95% CI, 0.74–0.87) for moderate and 0.73 (95% CI, 0.61–0.87) for high (both *p* < 0.001); each 10-point higher LE8 was associated with OR 0.91 (95% CI, 0.89–0.94). WQS regression identified smoking, BMI, sleep health, and blood pressure as the major contributors, whereas blood glucose, diet quality, blood lipids, and physical activity contributed only modestly. Results were consistent across subgroup and sensitivity analyses.

**Conclusion:**

In this cross-sectional analysis, higher Life’s Essential 8 scores were associated with lower odds of impaired pulmonary function. However, women comprised only 385 (2.2%) of the sample; thus, the findings primarily reflect a male cohort and may not be generalizable to women.

## 1 Introduction

Pulmonary function impairment not only reflects underlying respiratory disease but also serves as a biomarker of systemic health status within specific demographic contexts. Evidence indicates that reduced pulmonary function independently predicts both respiratory and cardiovascular diseases (1). Even declines within clinically normal ranges are associated with significantly elevated risks of mortality and cardiovascular events (2). Multicenter studies have further demonstrated that reductions in FEV₁ and FVC correspond to a graded increase in all-cause mortality and cardiovascular disease incidence. For example, the PURE cohort across 17 countries reported that each mild-to-moderate decrease in FEV₁ was associated with a marked increase in all-cause mortality and cardiovascular event risk (3). Similarly, a large Chinese cohort study found that both restrictive and obstructive pulmonary function abnormalities were significantly linked to higher risks of multiple cardiovascular events (4). Analyses from the Gutenberg cohort further indicated that, even after accounting for cardiac function, FEV₁ and FVC remained significant predictors of all-cause mortality (2). Collectively, these findings suggest that even mild reductions in lung function can independently forecast the risk of cardiovascular and systemic diseases (5, 6).

Traditional cardiovascular disease (CVD) risk factors—including poor diet, insufficient physical activity, smoking, obesity, hypertension, and hyperglycemia—also impact pulmonary health. In 2010, the American Heart Association proposed the “Life’s Simple 7” (LS7) metric, which integrates these seven factors to assess cardiovascular health (7). In 2022, this metric was updated to the “Life’s Essential 8” (LE8), adding healthy sleep as an eighth dimension and extending the scoring range for each component to 0–100 (8). Multiple studies have confirmed that higher composite cardiovascular health (CVH) scores are associated with lower risks of cardiovascular events and all-cause mortality (9). For instance, the Kailuan study in China observed a graded association among young adults aged 18–40, where low CVH scores were linked to substantially increased risks of early-onset cardiovascular disease and all-cause mortality (10).

Importantly, cardiovascular and respiratory health are intrinsically interconnected. Chronic respiratory diseases and cardiovascular diseases share common risk factors and frequently co-occur (11). Recent studies have begun to employ LE8 as a comprehensive metric to examine cardiopulmonary health relationships. Analyses based on NHANES data demonstrated a significant positive correlation between LE8 total score and FEV₁ Z-scores (12), with each 10-point increase in LE8 score associated with a notable reduction in COPD risk (13). Additionally, large-scale cohort studies in the United Kingdom indicated that both restrictive and obstructive lung function impairments substantially increased the risk of future cardiovascular events (adjusted HRs approximately 1.8 and 1.7, respectively) (14). These findings suggest that optimizing physical activity, body weight, blood pressure, and blood glucose—key components of cardiovascular health—may not only reduce cardiovascular risk but also synergistically support pulmonary function.

Despite these insights, research exploring the relationship between LE8 and lung function in the specific Chinese occupational cohorts remains limited. While prior studies have linked cardiovascular health indices to diabetes and cardiovascular and cerebrovascular diseases (15, 16), analyses specifically focusing on impaired pulmonary function are scarce. To address this gap, the present study utilized lung function data from over 17,000 adults in the Chinese Kailuan study to evaluate the association between LE8 composite scores and pulmonary function impairment. This investigation aims to advance understanding of cardiopulmonary health within this observed cohort and provide empirical evidence to inform integrated cardiopulmonary intervention strategies (17).

## 2 Methods

### 2.1 Study population and design

The Kailuan Study is a large-scale, ongoing prospective cohort established in the Kailuan community of Tangshan, China, with the aim of identifying potential determinants of human health. The cohort comprises all active and retired employees of the Kailuan Group, who are invited to participate in comprehensive biennial health assessments. Each assessment includes a standardized questionnaire, detailed clinical examinations, and laboratory testing (18–20). The study protocol has been previously described in detail. The study was approved by the Ethics Committee of Kailuan General Hospital (approval no. 2006–05) in accordance with the Declaration of Helsinki, and all participants provided written informed consent. The design and conduct of the study adhere to the Strengthening the Reporting of Observational Studies in Epidemiology (STROBE) guidelines for cohort studies.

**Figure 1:**
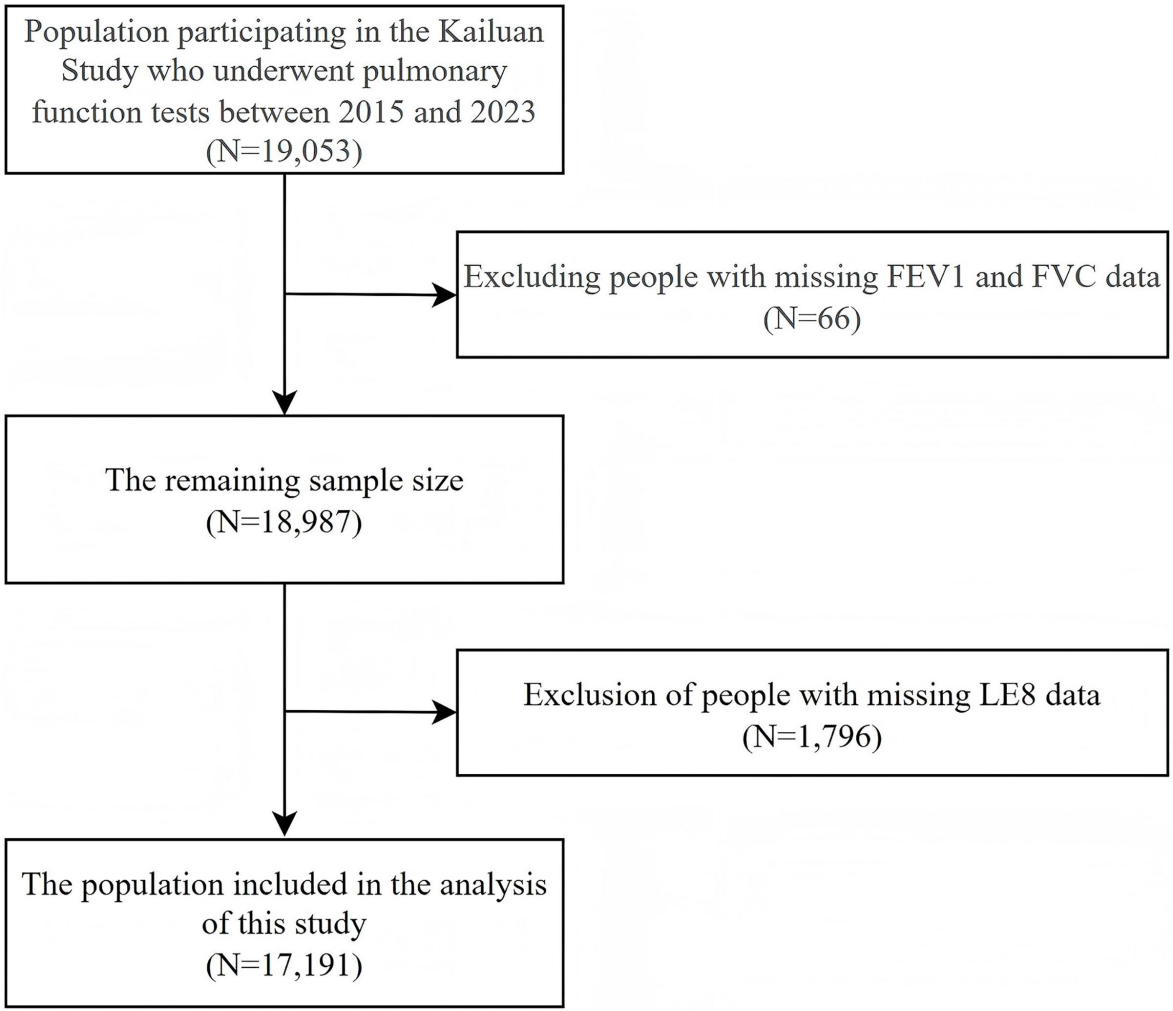
The flowchart illustrating the inclusion and exclusion process.

For the present analysis, we included participants who underwent pulmonary function testing between 2015 and 2023 (*n* = 19,053), as pulmonary function test was not incorporated into the Kailuan Study before 2015. Pulmonary function test was performed by trained technicians using calibrated specialized instrument in accordance with standardized operating procedures and predefined quality-control criteria. Participants were excluded if they lacked essential pulmonary function values (FEV₁ or FVC; *n* = 66) or had incomplete data required to calculate the cardiovascular health metric, Life’s Essential 8 (LE8; *n* = 1,796). After these exclusions, the final analytic cohort comprised 17,191 participants. Figure 1 provides a detailed participant flow diagram illustrating the exclusion process.

### 2.2 Assessment of life’s essential 8

Cardiovascular health (CVH) was assessed using the American Heart Association’s (AHA) Life’s Essential 8 (LE8) framework, which includes eight metrics categorized into two domains: health behaviors (diet, physical activity, nicotine exposure, and sleep health) and health factors (body mass index [BMI], blood lipids, blood glucose, and blood pressure) (8).

All clinical evaluations were conducted by trained healthcare staff following standardized protocols. Information on health behaviors and medication use was obtained through structured questionnaires. Dietary health was evaluated based on self-reported intake of salt, high-fat foods, and tea, reflecting dietary patterns previously linked to cardiovascular disease (CVD) risk in the Chinese population (21, 22). Anthropometric measurements included height and weight, obtained with participants wearing light indoor clothing and no shoes, to calculate BMI. Blood pressure was measured three times after a 5-min seated rest using a calibrated sphygmomanometer, and the mean of the three readings was used in analyses. Venous blood samples were collected after an overnight fast of 8–10 h and analyzed at a central laboratory with a Hitachi 747 automated analyzer (Hitachi, Tokyo, Japan) (23). Fasting blood glucose was measured using the hexokinase/glucose-6-phosphate dehydrogenase method. Total cholesterol and high-density lipoprotein cholesterol (HDL-C) were determined enzymatically, and non-high-density lipoprotein cholesterol (non-HDL-C) was calculated as total cholesterol minus HDL-C (24).

Following the AHA scoring algorithm, each of the eight metrics was assigned a continuous score from 0 to 100. The overall CVH score was calculated as the unweighted mean of the eight component scores. With the exception of BMI, scoring criteria followed AHA recommendations (e.g., sleep duration of 7 to <9 h per night scored 100). For BMI, modified cut-off points were applied (e.g., <23.0 kg/m^2^ scored 100) in accordance with the World Health Organization (WHO) expert consultation criteria for Asian populations (25). Detailed scoring criteria for each metric are provided in Supplementary Table S1. Based on the overall score, CVH status was categorized as high (80–100), moderate (50–79), or low (0–49).

### 2.3 Assessment of spirometry

Pulmonary function was assessed using a Japanese Minato AS-507 spirometer. The examination room temperature was maintained between 22 °C and 25 °C. Participants were instructed to avoid heavy meals and refrain from consuming cola, coffee, or tea within 2 h before the test, to abstain from smoking within 1 h, and to avoid vigorous physical activity within 30 min prior to testing. All procedures were performed under strict quality control, with professional guidance provided to ensure data accuracy. During the test, participants were required to sit upright without leaning against the chair back, keep both feet flat on the floor without crossing legs, and maintain the head in a neutral or slightly extended position while avoiding flexion. Forced vital capacity (FVC) and forced expiratory volume in 1 s (FEV1) were the primary parameters measured and recorded. After resting for 15 min in the seated position, participants wore a nose clip and performed at least three acceptable maneuvers that met grade A quality control standards; the best FEV1 curve was selected for analysis. If the three attempts did not meet the required standard, additional tests were conducted, not exceeding eight in total. The standards for pulmonary function tests are in accordance with the pulmonary function test guidelines issued by the Pulmonary Function Professional Group of the Respiratory Diseases Branch of the Chinese Medical Association (26), predicted percentages for FEV₁ (FEV₁% predicted) and FVC (FVC% predicted) were calculated using the Global Lung Function Initiative 2012 reference equations, which incorporate age, sex, height, and ethnicity (27).

### 2.4 Assessment of pulmonary function impairment

In this study, pulmonary function impairment (PFI) was defined as a forced expiratory FEV1 or FVC of less than 80% of the predicted value, in accordance with the Global Initiative for Chronic Obstructive Lung Disease (GOLD) 2023 guidelines and previous studies (28, 29).

### 2.5 Assessment of covariates

Covariates were selected with reference to prior relevant literature, and multiple imputation techniques were applied to handle missing data. Information on covariates, including Age, Gender, Alcohol drinking (current vs. non-current), education level (High school or above vs. below High school), and average monthly income per family member (<3,000 CNY vs. ≥3,000 CNY), was collected using a structured questionnaire.

### 2.6 Statistical analysis

Descriptive statistics were stratified by LE8 score categories (Low [0–49], Moderate [50–79], High [80–100]). Continuous variables are expressed as mean ± standard deviation and compared across groups using one-way analysis of variance (ANOVA). Categorical variables are presented as counts and percentages and compared using the Pearson *χ*^2 test.

Multivariable logistic regression was employed to examine the association between LE8 (including overall score, health behavior score, and health factor score) and pulmonary function impairment. Odds ratios (ORs) with 95% confidence intervals (CIs) were calculated for LE8 categories, using the low-score group as the reference, as well as for each 10-point increase in score. Three models were specified: Model 1 (unadjusted), Model 2 (adjusted for age and gender) and Model 3 (adjusted for age, Gender, Personal monthly income ≥3,000 CNY, High school or above and alcohol drinking).

For each LE8 domain (overall, behavior, and factor scores), the population attributable risk percentage (PAR%) for pulmonary impairment and its 95% CI were calculated. PAR% quantifies the proportion of pulmonary impairment within the studied sample attributable to suboptimal cardiovascular health (i.e., lower LE8 scores). In this cross-sectional study, PAR% was estimated from adjusted ORs using standard epidemiological methods.

Subgroup analyses were conducted by stratifying participants according to age (<40 vs. ≥40 years), gender (men vs. women), alcohol drinking (current vs. non-current), educational level (high school or above vs. below high school), and income (≥3,000 CNY vs. <3,000 CNY). Within each stratum, adjusted logistic regression models were fitted to estimate the association between LE8 score and pulmonary impairment. Interaction terms between LE8 score and each stratification variable were included to formally test for effect modification, with corresponding *p* values reported.

Restricted cubic spline (RCS) regression was applied to evaluate potential non-linear dose–response relationships between continuous LE8 scores and pulmonary impairment. In this model, LE8 scores were entered as spline terms with pre-specified knots, allowing for flexible modeling of the log-odds of impairment across the score distribution. This approach facilitates the detection and visualization of non-linear associations in logistic regression.

To evaluate the relative contributions of individual LE8 components, weighted quantile sum (WQS) regression was applied. This method estimates the combined effect of correlated exposures while simultaneously assigning weights that reflect the relative importance of each component.

Sensitivity analyses were conducted to evaluate the robustness of the findings. Analyses were repeated after excluding participants with a history of cardiovascular disease, lung disease and cancer to minimize potential residual confounding.

All statistical tests were two-sided, and *p* values <0.05 were considered statistically significant. Analyses were performed using SAS software, version 9.4 (SAS Institute, Cary, NC), and R software, version 4.2.2 (R Foundation for Statistical Computing, Vienna, Austria).

## 3 Results

### 3.1 Baseline characteristics

A total of 17,191 participants were included in the analysis (Table 1). Of these, 3,395 (19.7%) were classified as having low cardiovascular health (LE8 score 0–49), 13,069 (76.0%) as moderate (LE8 score 50–79), and 727 (4.2%) as high (LE8 score 80–100). The mean age of the cohort was 43.6 ± 9.5 years, with significant differences across LE8 categories (low: 46.1 ± 8.9; moderate: 43.2 ± 9.5; high: 39.3 ± 8.7; *p* < 0.001). The population was predominantly male (16,806/17,191; 97.8%); however, the proportion of women increased with higher LE8 scores (low: 9/3,395 [0.3%]; moderate: 303/13,069 [2.3%]; high: 73/727 [10.0%]; *p* < 0.001).

**Table 1:** Baseline characteristics.

Characteristic	LE8 score group				*p*-value^2^
	Overall *N* = 17,191^1^	L (0–49) *N* = 3,395^1^	M (50–79) *N* = 13,069^1^	H (80–100) *N* = 727^1^	
Age, years	43.6 ± 9.5	46.1 ± 8.9	43.2 ± 9.5	39.3 ± 8.7	<0.001
Gender, n (%)					<0.001
M	16,806 (97.8%)	3,386 (99.7%)	12,766 (97.7%)	654 (90.0%)	
F	385 (2.2%)	9 (0.3%)	303 (2.3%)	73 (10.0%)	
High school or above, n (%)					0.012
Y	8,095 (47.1%)	1,532 (45.1%)	6,197 (47.4%)	366 (50.3%)	
N	9,096 (52.9%)	1,863 (54.9%)	6,872 (52.6%)	361 (49.7%)	
Personal monthly income ≥3,000 CNY, n (%)					0.010
Y	4,086 (23.8%)	803 (23.7%)	3,144 (24.1%)	139 (19.1%)	
N	13,105 (76.2%)	2,592 (76.3%)	9,925 (75.9%)	588 (80.9%)	
Alcohol drinking, n (%)					<0.001
Y	6,153 (35.8%)	1,515 (44.6%)	4,430 (33.9%)	208 (28.6%)	
N	11,038 (64.2%)	1,880 (55.4%)	8,639 (66.1%)	519 (71.4%)	
LE8 score	60.2 ± 11.8	43.3 ± 5.4	63.3 ± 7.8	84.0 ± 3.4	<0.001
Health behaviors score	51.9 ± 16.4	35.5 ± 12.4	54.7 ± 13.9	77.4 ± 9.2	<0.001
Diet quality score	38.2 ± 19.8	34.3 ± 19.9	38.7 ± 19.5	47.5 ± 21.2	<0.001
Physical activity score	31.2 ± 38.5	15.2 ± 27.9	33.0 ± 38.9	73.7 ± 33.4	<0.001
Smoking score	54.6 ± 47.9	20.6 ± 38.4	61.2 ± 46.7	94.1 ± 21.0	<0.001
Sleep health score	83.6 ± 25.0	72.1 ± 31.7	86.0 ± 22.4	94.4 ± 12.5	<0.001
Health factors score	68.5 ± 16.7	51.0 ± 12.8	71.8 ± 14.3	90.7 ± 8.7	<0.001
BMI score	66.3 ± 24.3	52.8 ± 21.6	68.6 ± 23.6	87.8 ± 18.1	<0.001
Blood lipids score	71.0 ± 29.0	49.1 ± 27.1	75.4 ± 27.0	94.7 ± 14.9	<0.001
Blood glucose score	83.6 ± 24.0	68.6 ± 28.1	86.7 ± 21.6	97.6 ± 9.9	<0.001
Blood pressure score	53.1 ± 29.4	33.5 ± 23.9	56.6 ± 28.2	82.6 ± 23.5	<0.001
Pulmonary function impairment, n (%)					<0.001
Y	5,743 (33.4%)	1,311 (38.6%)	4,227 (32.3%)	205 (28.2%)	
N	11,448 (66.6%)	2,084 (61.4%)	8,842 (67.7%)	522 (71.8%)	

1 Mean ± SD; n (%).

2 One-way analysis of means; Pearson’s Chi-squared test.

LE8, Life’s Essential 8. BMI, body mass index.

Higher LE8 scores were also associated with more favorable sociodemographic and lifestyle characteristics. Educational attainment was greater among those with higher scores, with 8,095 participants (47.1%) overall having completed high school or above (high LE8: 50.3% vs. moderate: 47.4% vs. low: 45.1%; *p* = 0.012). Alcohol consumption declined progressively across categories (overall: 35.8%; low: 44.6% vs. moderate: 33.9% vs. high: 28.6%; *p* < 0.001). Monthly income ≥3,000 CNY was reported by 4,086 participants (23.8%) and differed modestly among groups *p* = 0.010. Pulmonary function impairment was common (5,743/17,191; 33.4%) and showed an inverse gradient with LE8 scores, decreasing from 38.6% in the low group to 32.3% in the moderate group and 28.2% in the high group (*p* < 0.001).

### 3.2 Association between life’s essential 8 and pulmonary function impairment

The associations between the Life’s Essential 8 (LE8) score and pulmonary function impairment are presented in Table 2. After adjustment covariates (Model 3), a clear dose-dependent inverse relationship was observed between overall LE8 score and the odds of pulmonary function impairment. Compared with participants in the low cardiovascular health (CVH) group (score 0–49), those in the moderate (50–79) and high (80–100) groups had significantly lower odds of impairment, with odds ratios (ORs) of 0.80 (95% CI, 0.74–0.87; *p* < 0.001) and 0.73 (95% CI, 0.61–0.87; *p* < 0.001), respectively. When modeled continuously, each 10-point increment in LE8 score was associated with a 9% reduction in the odds of pulmonary function impairment (OR, 0.91; 95% CI, 0.89–0.94; *p* < 0.001).

**Table 2:** Association between the Life’s Essential 8 score and the risk of pulmonary function impairment.

Subgroup	LE8 score						P for trend	Per 10 points	*p*-value
	L (0–49)		M (50–79)		H (80–100)				
	OR (95% CI)	*p*-value	OR (95% CI)	*p*-value	OR (95% CI)	*p*-value			
LE8 score group									
Cases/Total	1,311/3,395		4,227/13,069		205/727				
Model 1	Reference	-	0.76 (0.70, 0.82)	<0.001	0.62 (0.52, 0.74)	<0.001	<0.001	0.88 (0.86–0.91)	<0.001
Model 2	Reference	-	0.81 (0.75, 0.88)	<0.001	0.75 (0.62, 0.89)	0.001	<0.001	0.92 (0.89–0.94)	<0.001
Model 3	Reference	-	0.80 (0.74, 0.87)	<0.001	0.73 (0.61, 0.87)	<0.001	<0.001	0.91 (0.89–0.94)	<0.001
PAR, %					15.5 (5.9, 24.5)	<0.001			

CI, Confidence Interval; OR, Odds Ratio. Model 1: no covariates were adjusted. Model 2: adjusted for Age and Gender. Model 3: adjusted for Age, Gender, Personal monthly income ≥3,000 CNY, High school or above and alcohol drinking.

After adjustment covariates, the Health Factors sub-score was independently and inversely associated with pulmonary function impairment, with no significant association observed for the Health Behaviors sub-score. Compared with participants in the low-scoring group, the ORs for the moderate and high Health Factors groups were 0.78 (95% CI, 0.71–0.86; *p* < 0.001) and 0.69 (95% CI, 0.62–0.77; *p* < 0.001).

Population attributable risk (PAR) analysis indicated that 15.5% (95% CI, 5.9–24.5%) of pulmonary function impairment cases in this cohort could be attributed to non-ideal (low or moderate) LE8 score, while non-ideal Health factors contributed 12.3% (95% CI, 8.7–15.5%) to the risk.

### 3.3 Subgroup analyses

**Figure 2:**
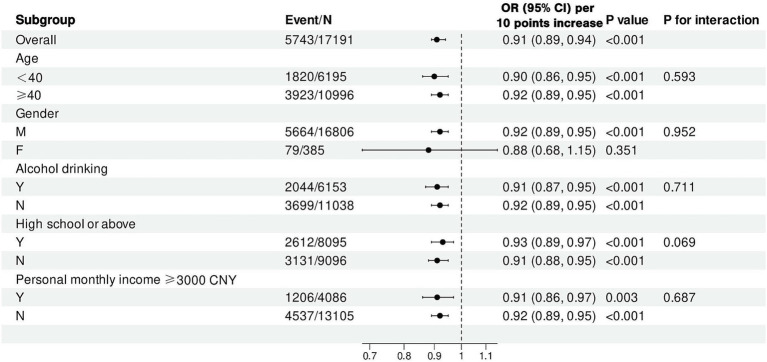
Subgroup Analyses of the association between a 10-point increase in Life’s Essential 8 score and pulmonary function impairment. OR adjusted for Age, Gender, Personal monthly income ≥3,000 CNY, High school or above and alcohol drinking.

**Figure 3:**
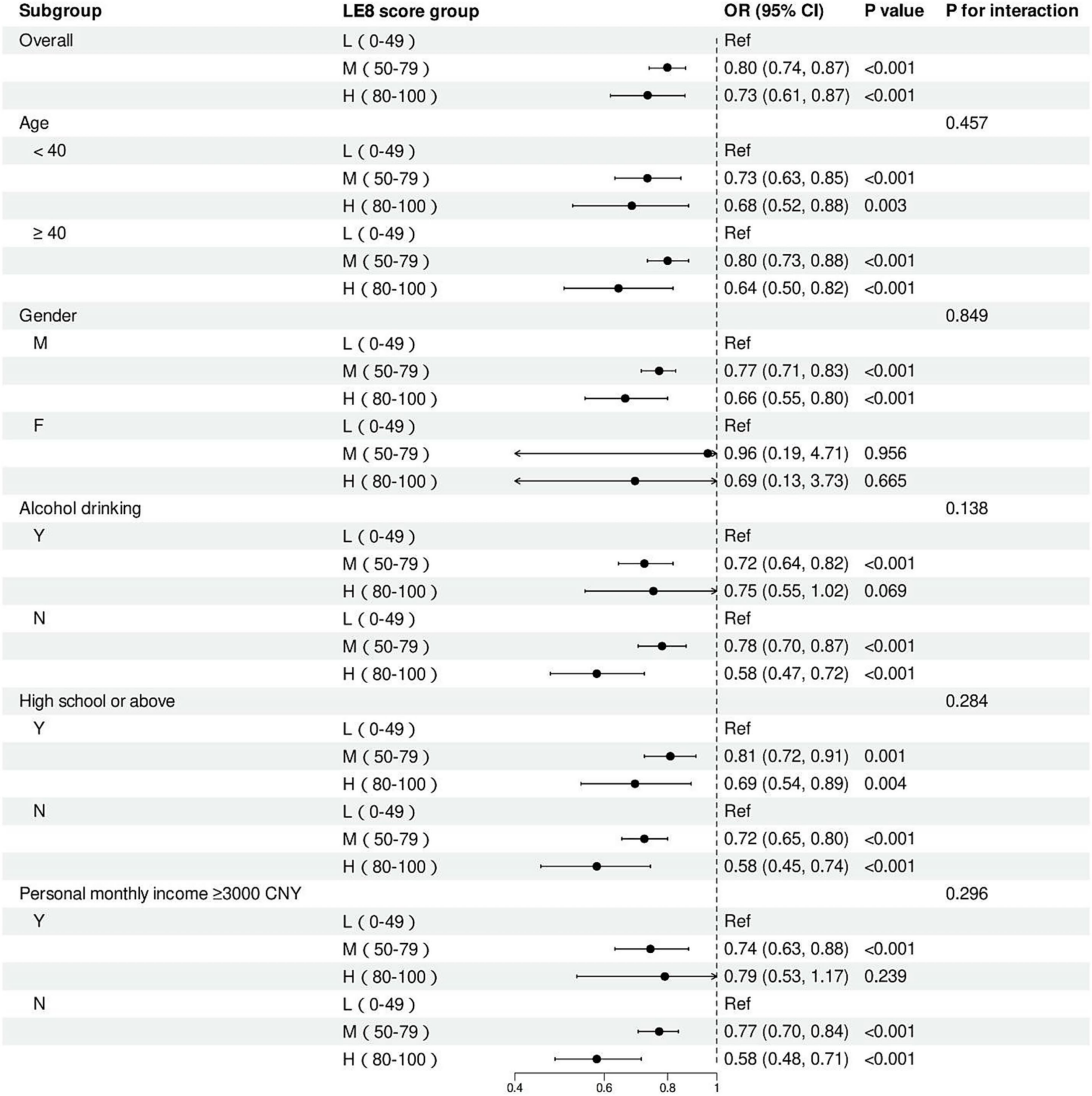
Subgroup analyses of Life’s Essential 8 score and the risk of pulmonary function impairment. OR adjusted for Age, Gender, Personal monthly income ≥3,000 CNY, High school or above and alcohol drinking.

Pre-specified subgroup analyses were performed by age, gender, alcohol consumption, education level, and personal monthly income to assess the consistency of the association between LE8 score and pulmonary function impairment (Figures 2, 3). When modeled as a continuous variable, each 10-point increase in LE8 score was consistently associated with lower odds of impairment across all strata. Formal interaction testing showed no evidence of effect modification by any stratification variable (all P for interaction > 0.05).

Compared with the low-LE8 group, the adjusted ORs for the high-LE8 group were 0.68 (95% CI, 0.52–0.88) among participants <40 years and 0.64 (95% CI, 0.50–0.82) among those ≥40 years. Similar robustness was observed across alcohol consumption, education level and income. When modeled continuously (Figure 2), each 10-point LE8 increment showed a similar protective trend in both men (OR 0.92; 95% CI 0.89–0.95, *p* < 0.001) and women (OR 0.88; 95% CI 0.68–1.15, *p* = 0.351), with no significant interaction by sex (P for interaction = 0.952). In categorical analyses (Figure 3), results for men closely aligned with the overall cohort (Moderate: OR 0.77, *p* < 0.001; High: OR 0.66, *p* < 0.001). For women, risk estimates suggested a consistent protective direction but generated imprecise, wide confidence intervals (Moderate: OR 0.96, 95% CI 0.19–4.71; High: OR 0.69, 95% CI 0.13–3.73). The imprecision of the estimates in females may be attributable to the relatively small number of female (*n* = 385, 2.2%) participants in the overall cohort.

### 3.4 Relative contribution of individual LE8 components to pulmonary function impairment

**Figure 4:**
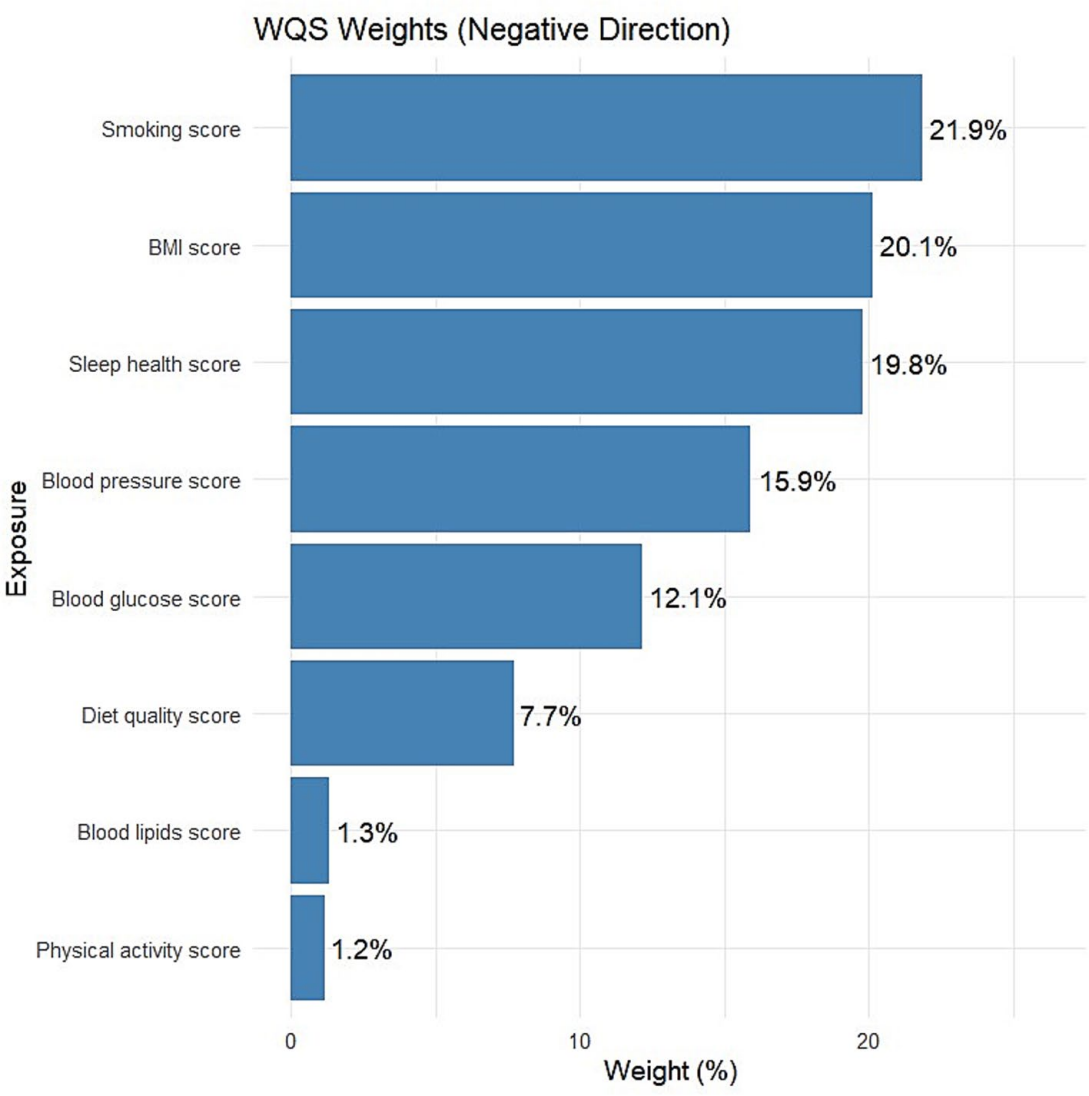
Contribution of Life’s Essential 8 components to pulmonary function impairment, estimated by WQS regression.

Weighted quantile sum (WQS) regression was applied to assess the relative contributions of individual Life’s Essential 8 (LE8) components to pulmonary function impairment (Figure 4). The WQS weights, which together totaled 100%, indicated that smoking was the predominant contributor (21.9%), followed by body mass index (BMI; 20.1%) and sleep health (19.8%). Blood pressure (15.9%) and blood glucose (12.1%) also made substantial contributions, whereas diet quality (7.7%), blood lipids (1.3%), and physical activity (1.2%) contributed to a lesser extent. These results suggest that, in this cohort, the inverse association between overall LE8 score and pulmonary function impairment was mainly driven by smoking, obesity (as reflected by BMI), and sleep health, while blood lipids and physical activity appeared to play comparatively minor roles.

### 3.5 Dose–response relationship between LE8 score and pulmonary function impairment

**Figure 5:**
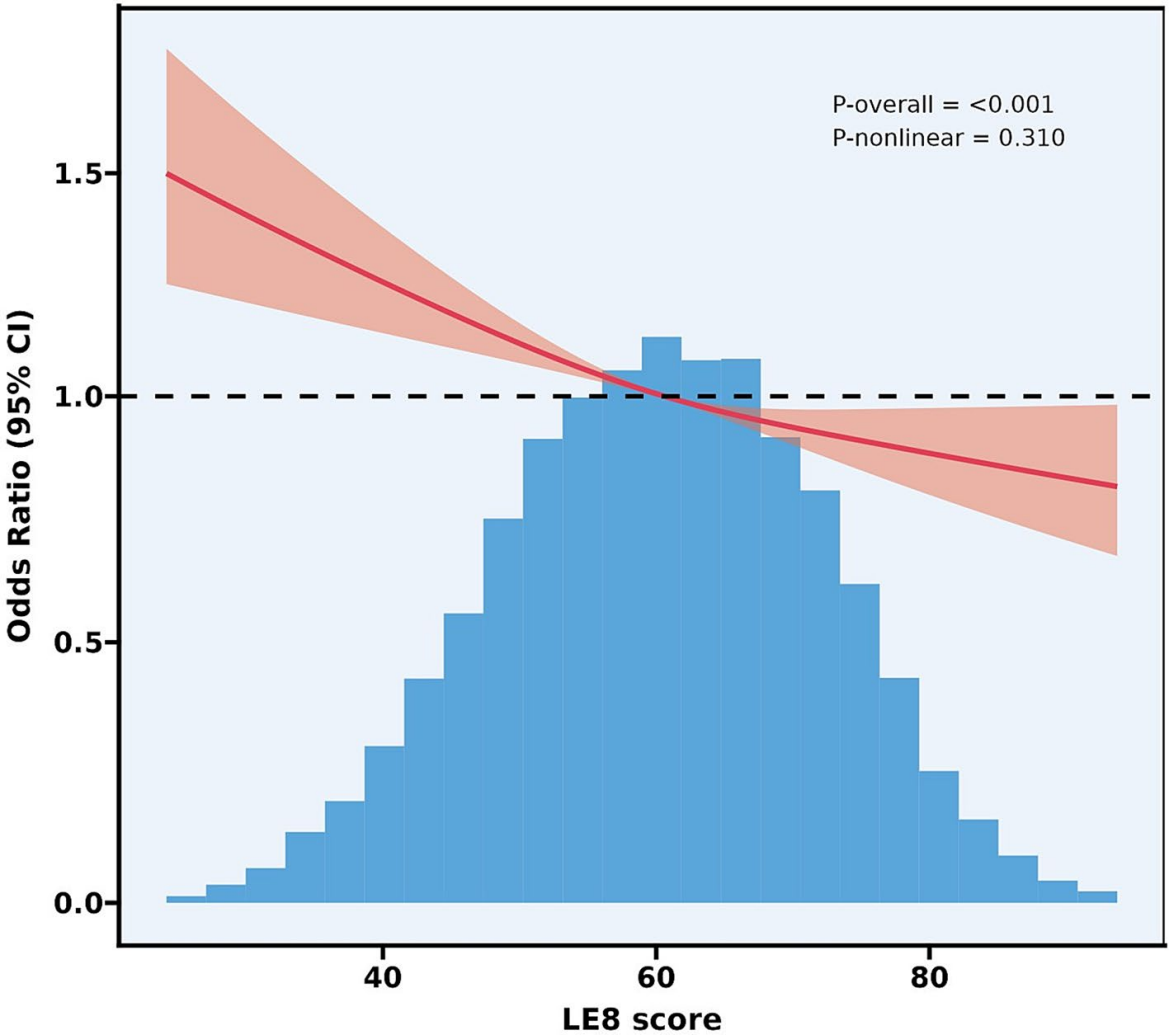
Dose–response relationship between the Life’s Essential 8 score and odds of pulmonary function impairment.

Restricted cubic spline (RCS) analyses—with four knots specified at the 5th, 35th, 65th, and 95th percentiles of the LE8 score distribution—demonstrated a linear inverse relationship between continuous LE8 scores and the odds of pulmonary function impairment (Figure 5). Higher LE8 scores were consistently associated with progressively lower odds of impairment across the score range.

### 3.6 Sensitivity analyses

Sensitivity analyses confirmed the robustness of our findings. Notably, even after strictly excluding participants with a prior history of cardiovascular disease, lung disease, or cancer to minimize potential reverse causation and residual confounding, the inverse association between higher LE8 categories and lower odds of pulmonary function impairment remained highly stable (Supplementary Table S3). These results strongly reinforce the overall reliability and robustness of our primary findings across different health statuses.

## 4 Discussion

In this large cohort of Chinese industrial workers, higher cardiovascular health, assessed using Life’s Essential 8 scores, was strongly and dose-dependently associated with lower odds of pulmonary function impairment. Compared with the low-CVH category, the odds of PFI were 20% lower in the moderate-CVH group and 27% lower in the high-CVH group. Similarly, each 10-point increment in the LE8 score corresponded to a 9% reduction in the odds of PFI. The clinical implications for similar demographic groups are notable: an estimated 15.5% of PFI cases in this specific cohort could be attributed to suboptimal CVH. Weighted contribution analyses identified smoking, elevated body mass index, and poor sleep health as the most influential contributors to pulmonary function impairment risk, followed by elevated blood pressure and abnormal blood glucose. In contrast, diet quality, blood lipids, and physical activity showed smaller but still statistically significant effects. Together, these findings underscore a strong systemic link between cardiovascular and respiratory health and support the LE8 metric as a practical tool for assessing integrated cardiorespiratory health.

Our findings align with prior studies that have linked composite cardiovascular health (CVH) metrics to respiratory outcomes. Earlier epidemiological work on Life’s Simple 7 (LS7) provides key context. In a cross-sectional analysis of US adults, Fan et al. reported that higher LS7 scores were linearly associated with better FEV1 and FVC values and with substantially lower odds of chronic obstructive pulmonary disease (COPD). Notably, current smoking and hypertension—two LS7 components—together accounted for approximately 21–22% of COPD risk (30). Similarly, the Multi-Ethnic Study of Atherosclerosis (MESA) showed that individuals with optimal LS7 scores, compared with those with inadequate scores, had markedly lower incidence of multiple chronic diseases, including COPD (hazard ratio ≈0.51) (31). Analyses from the National Health and Nutrition Examination Survey (NHANES) further demonstrated that higher LE8 scores were positively associated with FEV1 and FVC and inversely associated with both COPD prevalence and preserved ratio impaired spirometry (PRISm) (32–34). Collectively, these findings suggest that adults with more favorable CVH profiles tend to maintain better lung function and face a lower burden of respiratory disease (35).

The principal novelty of our study lies in validating the association between LE8 and pulmonary function impairment (PFI) within a large, prospective East Asian cohort. Most prior investigations of cardiovascular health (CVH) metrics have been conducted in North American and European populations (36, 37), where the burden of cardiovascular and respiratory diseases, as well as the relative impact of risk factors such as body mass index (BMI), differ substantially from those observed in East Asia (38). By confirming this relationship in the well-characterized Kailuan study (39), our findings address an important knowledge gap and strengthen evidence for the cross-cultural applicability of the LE8 framework.

Mechanistically, the concordance between CVH and lung health is biologically plausible given their shared risk factors and overlapping pathophysiological pathways. Each LE8 component—diet quality, physical activity, nicotine exposure, sleep health, BMI, blood pressure, blood lipids, and blood glucose—has the potential to influence pulmonary physiology. For example, obesity, reflected in the BMI component, directly impairs lung mechanics: excess adiposity restricts diaphragmatic excursion and chest wall compliance while amplifying systemic inflammation, leading to reductions in lung volumes and airflow (39). Similarly, metabolic disturbances captured by the LE8 framework exert well-established pulmonary effects. Diabetes is independently associated with restrictive ventilatory patterns; a large meta-analysis showed that individuals with type 2 diabetes had significantly lower FEV1 and FVC compared with non-diabetic peers, with mean deficits of approximately 7–9% predicted (40). Consistent with this evidence, our results demonstrate that participants with healthier glucose and lipid profiles had lower risks of pulmonary impairment, echoing prior findings that hyperlipidemia increases the risk of incident COPD by nearly 50% (41). In contrast, adherence to a diet rich in antioxidants—characterized by fruits, vegetables, and whole grains—has been shown to preserve lung integrity. Notably, adherence to high-quality dietary patterns such as the DASH diet is associated with reduced COPD prevalence and superior spirometric indices (42).

Similarly, several behavioral components of LE8 exert direct influences on pulmonary health. Regular physical activity has been shown to attenuate lung function decline, primarily through improvements in cardiopulmonary fitness and reductions in systemic inflammation (12). Evidence from both clinical trials and prospective cohorts demonstrates that higher levels of exercise or greater cardiorespiratory fitness are associated with slower declines in FEV1 and superior peak expiratory flow in older adults, potentially mediated by enhanced pulmonary repair capacity and increased antioxidant defenses (35). Sleep health—newly incorporated into the LE8 framework—also appears to be a critical determinant of respiratory outcomes. Both short (<6 h) and long (≥9 h) sleep durations have been associated with impaired lung function and elevated odds of restrictive ventilatory patterns in adults (43). In contrast, “ideal” sleep duration of approximately 7–8 h per night may help preserve lung volumes by supporting immune balance and limiting systemic inflammation. Among all LE8 factors, smoking (nicotine exposure) remains the most significant modifiable risk determinant of impaired lung health and COPD. As expected, the LE8 smoking component demonstrated a strong inverse association with pulmonary function in our analysis. Chronic tobacco exposure induces persistent airway inflammation, alters innate immune responses, and promotes airway remodeling and parenchymal destruction—pathological processes that define COPD^35^, these shared etiologic pathways provide a compelling rationale for why composite CVH metrics so closely track with pulmonary outcomes. Chronic lung and cardiovascular diseases frequently coexist; meta-analyses indicate that patients with COPD have approximately two- to threefold higher odds of coronary artery disease, heart failure, and stroke compared with the general population (44). These comorbidities are interconnected through systemic inflammation, oxidative stress, and hypoxemia. Impaired vascular function can reduce pulmonary perfusion, while obstructive lung disease imposes hemodynamic strain on the heart. Accordingly, our findings suggest that improving an individual’s LE8 profile—through smoking cessation, weight management, dietary optimization, and regular physical activity—may simultaneously enhance vascular health and preserve pulmonary function by mitigating shared inflammatory and metabolic stressors.

The WQS analysis demonstrated that the principal negative contributors to lung function were smoking (21.9%), elevated BMI (20.1%), poor sleep health (19.8%), and hypertension (15.9%), followed by smaller contributions from elevated blood glucose (12.1%) and unhealthy diet (7.7%). Smoking introduces oxidants and irritants into the distal airways and alveoli, generating reactive oxygen species that damage epithelial cells and surfactant, thereby eliciting chronic neutrophilic and eosinophilic inflammation (45). Such injury promotes airway remodeling and loss of elastic recoil, leading to reductions in FEV₁ and FVC. Obesity impairs ventilation mechanically through the accumulation of excess adipose tissue on the chest wall and abdomen, thereby reducing lung compliance. In addition, adipose tissue secretes pro-inflammatory adipokines (e.g., IL-6, CRP) and induces insulin resistance, systemic alterations that have been empirically linked to lower FEV₁ and FVC with a restrictive pattern (46). Likewise, inadequate or fragmented sleep contributes to systemic inflammation, as reflected by increased IL-6 and CRP levels, and has been associated with declines in FEV₁ and FVC (43). Chronic hypertension results in endothelial dysfunction and vascular remodeling within the pulmonary circulation, with hypertensive individuals exhibiting significantly reduced FEV₁ and FVC compared with normotensive controls (46). Elevated blood glucose exerts a modest influence on pulmonary decline; each 1 mmol/L increase in fasting glucose is associated with small reductions in FEV₁ and FVC (47), likely reflecting diabetes-related microvascular injury, oxidative stress, and advanced glycation that increase lung stiffness. Diets high in processed foods and saturated fat but low in antioxidants may further intensify oxidative stress and inflammation, aggravating lung tissue damage (48). In contrast, dyslipidemia and physical inactivity had minimal WQS weights (1.3 and 1.2%), suggesting relatively limited direct effects on spirometric indices within our cohort.

The comparatively weaker associations observed for diet and physical activity in our WQS model warrant a comprehensive pathophysiological and methodological interpretation. First, the specific occupational context of the Kailuan cohort is crucial. As a predominantly industrial and mining workforce, cumulative environmental and occupational exposures (e.g., coal dust and silica) serve as dominant drivers of pulmonary impairment, which may largely overshadow the more subtle, long-term protective effects of diet and physical activity. Second, physiological threshold effects likely exist; while moderate exertion benefits general cardiovascular health, significant spirometric improvements in FEV1 and FVC often require higher intensities and volumes of exercise. Third, measurement limitations inherently attenuate these associations. Our simplified dietary metric lacked data on antioxidant-rich components like fresh fruits and vegetables, and self-reported physical activity is subject to recall bias. Such non-differential misclassification typically biases risk estimates toward the null. Finally, our physical activity assessment did not differentiate between leisure-time physical activity (LTPA) and occupational physical activity (OPA). In blue-collar workers, high OPA is frequently associated with adverse health outcomes and systemic inflammation—a well-documented phenomenon known as the physical activity paradox (49, 50). This unmeasured confounding likely diluted the protective cardiovascular and respiratory signals of exercise in this specific demographic.

Furthermore, our analysis revealed an apparent statistical paradox between the WQS results and the sub-domain regression analysis for Health Behaviors (Supplementary Table S2). While WQS identified smoking and sleep health as leading contributors to pulmonary function impairment, the aggregate Health Behaviors sub-score showed no significant association in the fully adjusted model (OR 1.01, *p* = 0.883). This discrepancy stems from fundamental differences in score construction. The AHA LE8 sub-score is an unweighted arithmetic mean, inherently assuming equal theoretical contributions (25% each) from all four behavioral components. However, as noted above, the signals for diet and physical activity in our cohort were heavily attenuated due to measurement limitations and occupational confounding. Consequently, averaging the highly predictive signals of smoking and sleep with the low signals of diet and physical activity mathematically diluted the aggregate behavioral score, rendering it statistically non-significant. In contrast, the data-driven WQS approach successfully avoided this dilution by isolating the active components and assigning them weights based on their actual predictive strength, highlighting the necessity of evaluating specific behaviors rather than relying solely on unweighted aggregate metrics in complex occupational cohorts.

These findings have important clinical and public health implications. First, clinicians should recognize that optimal cardiovascular health provides measurable pulmonary benefits. Individuals with suboptimal LE8 profiles—such as uncontrolled blood pressure, dyslipidemia, or obesity—may be at increased risk not only for cardiovascular events but also for impaired lung function. Routine assessment of cardiovascular health could therefore facilitate the early identification of individuals at risk for pulmonary disease. Second, the estimated population-attributable risks indicate a substantial preventable burden: achieving ideal LE8 levels across highly susceptible occupational demographics could potentially prevent approximately one-fifth of lung function impairment cases, underscoring the value of integrated preventive strategies.

Despite the strengths of a large sample size and comprehensive cardiovascular health assessment, several limitations should be noted. First, the cross-sectional design precludes causal inference and does not allow for a temporal assessment of the relationship between cardiovascular health indicators and respiratory parameters. Crucially, the directionality of these associations remains uncertain, meaning reverse causation cannot be entirely ruled out. For instance, it is highly plausible that individuals with pre-existing pulmonary impairment experience reduced exercise tolerance and subsequent weight gain, which would concurrently lead to lower physical activity levels and suboptimal BMI, thereby reducing their overall LE8 score. Consequently, our findings should be considered hypothesis-generating. Future longitudinal and interventional studies are required to disentangle this complex relationship and evaluate whether improving LE8 components can directly prevent lung function decline. Second, several LE8 behavioral components—including diet, physical activity, and smoking—were assessed using self-administered questionnaires. This methodology inherently introduces recall bias and social desirability bias. From an epidemiological perspective, such non-differential misclassification typically biases risk estimates toward the null. This likely explains the attenuated associations and lower relative contributions observed for behavioral variables (e.g., WQS weights of 1.2% for physical activity and 7.7% for diet) compared to objectively measured health factors like BMI and blood pressure. Furthermore, our dietary evaluation metric was overly simplified. Due to the lack of detailed food frequency data in the historical Kailuan baseline, we utilized a surrogate index derived solely from the intake frequency of salt, fatty foods, and tea. While this specific 3-item proxy has been previously validated for cardiovascular risk assessment and widely utilized in established LE8 studies within the Chinese population (51, 52), its narrow scope omits critical components such as fresh fruits, vegetables, and whole grains, which confer protective antioxidant effects on respiratory health. Consequently, this simplified metric may have resulted in an underestimation of nutrition’s true impact on lung function. Future prospective studies would benefit from employing formally validated and culturally tailored instruments, such as the Physical Activity Vital Sign (PAVS) for exercise assessment and the KAP-HEQ for dietary evaluation (53, 54), to capture health behaviors more precisely. Third, a major limitation of this study is the markedly low representation of women (*n* = 385, 2.2%), which reflects the historical demographic composition of the Kailuan study and substantially limits the external validity of the findings. Although exploratory sex-stratified analyses were conducted, statistical power in the female subgroup was severely constrained, resulting in imprecise estimates with wide confidence intervals; accordingly, significant associations were observed only in men. Notably, no significant interaction by sex was detected. Therefore, these findings should be interpreted with caution and not generalized to women, underscoring the need for future studies with more balanced sex representation. Fourth, associations with pulmonary impairment were assessed cross-sectionally at baseline; longitudinal studies would better clarify the temporal relationships between LE8 changes and lung function decline. Fifth, the LE8 score inherently overlaps with lung health metrics, particularly through the smoking component and BMI, so the observed associations may partly reflect this embedded relationship. Sixth, spirometry outcomes were dichotomized as “impairment”; analyses using continuous lung function measures or specific respiratory conditions (e.g., emphysema, asthma) could provide additional mechanistic insight. Finally, future prospective studies should incorporate cardiopulmonary exercise testing (CPET). As the gold standard for integrated cardiorespiratory evaluation, utilizing CPET-derived parameters (e.g., VO₂peak and ventilatory efficiency indices) would provide deeper mechanistic insights into the associations between LE8 and lung function, thereby strengthening the clinical and translational relevance of these findings.

## 5 Conclusion

In this large cohort of predominantly male industrial workers, higher CVH as defined by LE8 is significantly associated with a lower prevalence of impaired pulmonary function. These findings highlight the systemic link between cardiovascular and respiratory health, even in a population with significant occupational risk factors. LE8 may be a useful tool for integrated health assessment in this specific demographic. These findings highlight the shared pathophysiology of cardiovascular and pulmonary diseases and underscore the potential for integrated prevention strategies. Emphasizing specific LE8 metrics—particularly tobacco avoidance, adequate sleep, optimal body weight, and favorable blood pressure—may confer dual benefits for vascular and respiratory health. However, as our PAR% analysis indicated that modifying non-smoking behavioral components in aggregate did not statistically reduce the PFI burden, public health initiatives in similar occupational cohorts should prioritize smoking cessation and clinical factor control over generalized lifestyle interventions. Targeted health initiatives might leverage this overlap, as promoting adherence to LE8 components may support simultaneous reductions in the burden of cardiovascular and chronic lung disease in similar cohort settings, providing a unified target for healthy aging across organ systems.

## Data Availability

The raw data supporting the conclusions of this article will be made available by the authors, without undue reservation.
