# Supplementary material for: Association of life’s essential 8 with pulmonary function impairment: a cross-sectional analysis of the Kailuan study
Source: Front Nutr. 2026 May 28;13:1750094. doi: 10.3389/fnut.2026.1750094 (PMC13253279; doi:10.3389/fnut.2026.1750094)
Supplement: Supplementary file 1 [file Table_1.DOCX]

Supplementary Material

**Table S1.** Definition and scoring approach for quantifying cardiovascular health in the Kailuan study (Life’s Essential 8 Kailuan Version)

| Domain | Metric | Method of measurement | Quantification of CVH metric |
| --- | --- | --- | --- |
| Health behaviors | Diet health | Measurement: Self-reported intake of salt, fatty foods, and tea  Examples of salt intake measurement: “What flavor do you prefer.”  Examples of fatty food intake measurement: “How often do you eat fatty foods? ”  Example of salt intake measurement: “How often do you drink tea?” | Metric: The unweighted average of salt, fatty food, and tea scoring.  Salt scoring:  Points Level  100 <6 g/day  50 6-12 g/day   1. >12 g/day   Fatty food scoring:  Points Level  100 <1 time/week  50 1-3 times/week   1. >3 times/week   Tea scoring:  Points Level   1. ≥4 times/week   75 1-3 times/week  50 1-3 times/month  25 < 1 time/month  0 Never |
|  | Physical activity | Measurement: Self-reported times of physical activity per week.  Example tools for measurement: “How many times did you usually spend on physical activity (note: It took at least 20 minutes each time)? ” | Metric: Minutes of physical activity per week.  Scoring:  Points Level  100 ≥ 80  50 20-60  0 <20 |
|  | Nicotine exposure | Measurement: Self-reported use of cigarettes  Example tools for measurement: Do you now smoke cigarettes? (Never smoker, former smoker, some days, every day) | Metric: Smoking status  Scoring:  Points Status  100 Never smoker  50 Former smokers quit ≥ 1 y  25 Current smokers,< 1cigarette/d  0 Current smoker, ≥1cigarette/d |
|  | Sleep health | Measurement: Self-reported average hours of sleep per night  Example tools for measurement: “On average, how many hours of sleep do you get per night?” | Metric: Average hours of sleep per night  Scoring:  Points Level  100 7 - <9 h  90 9 - < 10 h  70 6 - < 7 h  40 5 - <6 or ≥10 h  20 4 - <5 h  0 <4h |
| Health factors | Body mass index | Measurement: Body weight (kg) divided by height squared(m²)  Example tools for measurement: Objective measurement of height and weight | Metric: Body mass index (kg/m^2^)  Scoring:  Points Level  100 <23  75 23.0-24.9  50 25.0-29.9  25 30.0-34.9  0 ≥35.0 |
|  | Blood lipids | Measurement: Plasma total and HDL cholesterol with the calculation of non-HDL cholesterol.  Example tools for measurement: Fasting blood sample.  non-HDL-cholesterol unit conversion:  1mmol/L= 38.67mg/L  1mg/L=0.02586mmol/L | Metric: Non-HDL cholesterol (mmol/L)  Scoring:  Points Level  100 < 3.36  60 3.36-4.13  40 4.14-4.90  20 4.91-5.68  0 ≥ 5.69  If the drug-treated level subtracts 20 points |
|  | Blood glucose | Measurement: Fasting blood glucose(FBG)  Example tools for measurement: Fasting blood glucose sample.  HBA1C to FBG(mg/L) to conversion:  28.7 * A1C - 46.7 = FBG  FBG unit conversion:  1mg = 0.056mmol/L  1mmol/L= 18.02 mg/dL | Metric: FBG (mmol/L)  Scoring:  Points Level  100 No history of diabetes with FBG <5.6  60 No diabetes with FBG 5.6-6.9  40 Diabetes with FBG < 8.6  30 Diabetes with FBG 8.6-10.1  20 Diabetes with FBG 10.2-11.6  10 Diabetes with FBG 11.7-13.2  0 Diabetes with FBG ≥13.3 |
|  | Blood pressure | Measurement: Appropriately measured systolic and diastolic blood pressure  Example tools for measurement: Corrected Mercury sphygmomanometer | Metric: Systolic and diastolic blood pressure (mm Hg)  Scoring:  Points Level  100 <120 / < 80  75 120-129 / < 80  50 130-139 or 80-89  25 140-159 or 90-99  0 ≥160 or ≥100  Subtract 20 points if the treated level |

| **Table S2. Associations of the Health behaviors score and Health factors group with the risk of pulmonary function impairment.** | | | | | | | | | |
| --- | --- | --- | --- | --- | --- | --- | --- | --- | --- |
| **Subgroup** | **LE8 score** | | | | | | **P for trend** | **Per 10 points** | **P-value** |
|  | **Low CVH (0–49)** | | **Moderate CVH (50–79)** | | **High CVH (80–100)** | |  |  |  |
|  | **OR (95% CI)** | **P-value** | **OR (95% CI)** | **P-value** | **OR (95% CI)** | **P-value** |  |  |  |
| **Health behaviors group** |  | | | | | | | | |
| Cases/Total | 2,384/ 6,898 | | 3,046/ 9,359 | | 313/ 934 | |  | | |
| Model 1 | Reference | - | 0.91 (0.86, 0.98) | 0.007 | 0.95 (0.83, 1.10) | 0.527 | 0.028 | 0.98 (0.96-0.99) | 0.013 |
| Model 2 | Reference | - | 0.93 (0.87, 1.00) | 0.044 | 1.02 (0.88, 1.18) | 0.792 | 0.221 | 0.99 (0.97-1.01) | 0.171 |
| Model 3 | Reference | - | 0.93 (0.87, 1.00) | 0.046 | 1.01 (0.87, 1.17) | 0.883 | 0.204 | 0.99 (0.97-1.00) | 0.136 |
| Population attributable risk, % |  | | | | -0.31 (-8.5, 8.8) | 0.928 |  |  |  |
| **Health factors group** |  | | | | | | | | |
| Cases/Total | 974/ 2,397 | | 3,410/ 10,156 | | 1,359/ 4,638 | |  | | |
| Model 1 | Reference | - | 0.74 (0.67, 0.81) | <0.001 | 0.61 (0.55, 0.67) | <0.001 | <0.001 | 0.91 (0.89-0.92) | <0.001 |
| Model 2 | Reference | - | 0.78 (0.71, 0.86) | <0.001 | 0.70 (0.63, 0.77) | <0.001 | <0.001 | 0.93 (0.91-0.95) | <0.001 |
| Model 3 | Reference | - | 0.78 (0.71,0.86) | <0.001 | 0.69 (0.62, 0.77) | <0.001 | <0.001 | 0.93 (0.91-0.95) | <0.001 |
| Population attributable risk, % |  | | | | 12.3 (8.7, 15.5) | <0.001 |  | | |
| Abbreviations: CI = Confidence Interval, OR = Odds Ratio | | | | | | | | |  |
| Model 1 : no covariates were adjusted  Model 2 : adjusted for Age and Gender  Model 3 : adjusted for Age, Gender, Personal monthly income ≥3000 CNY, High school or above and alcohol drinking. | | | | | | | | |  |

| **Table S3.** **Sensitivity Analyses for the Association between Life's Essential 8 Score and Pulmonary Function Impairment.** | | | | | | | | | | | | | | | |
| --- | --- | --- | --- | --- | --- | --- | --- | --- | --- | --- | --- | --- | --- | --- | --- |
| **Characteristic** | **Model 1** | | | | | **Model 2** | | | | | **Model 3** | | | | |
|  | **N** | **Event N** | **OR** | **95% CI** | **p-value** | **N** | **Event N** | **OR** | **95% CI** | **p-value** | **N** | **Event N** | **OR** | **95% CI** | **p-value** |
| **Exclude individuals with CVD** |  |  |  |  |  |  |  |  |  |  |  |  |  |  |  |
| L（0-49） | 3,194 | 1,230 | — | — |  | 3,194 | 1,230 | — | — |  | 3,194 | 1,230 | — | — |  |
| M（50-79） | 12,698 | 4,061 | 0.75 | 0.69, 0.81 | <0.001 | 12,698 | 4,061 | 0.80 | 0.73, 0.86 | <0.001 | 12,698 | 4,061 | 0.79 | 0.73, 0.86 | <0.001 |
| H（80-100） | 724 | 205 | 0.63 | 0.53, 0.75 | <0.001 | 724 | 205 | 0.74 | 0.62, 0.89 | 0.001 | 724 | 205 | 0.73 | 0.61, 0.87 | <0.001 |
| P for trend |  |  |  |  | <0.001 |  |  |  |  | <0.001 |  |  |  |  | <0.001 |
| **Exclude individuals with lung disease** |  |  |  |  |  |  |  |  |  |  |  |  |  |  |  |
| L（0-49） | 3,388 | 1,305 | — | — |  | 3,388 | 1,305 | — | — |  | 3,388 | 1,305 | — | — |  |
| M（50-79） | 13,047 | 4,211 | 0.76 | 0.70, 0.82 | <0.001 | 13,047 | 4,211 | 0.81 | 0.75, 0.88 | <0.001 | 13,047 | 4,211 | 0.80 | 0.74, 0.87 | <0.001 |
| H（80-100） | 726 | 205 | 0.63 | 0.53, 0.75 | <0.001 | 726 | 205 | 0.75 | 0.63, 0.90 | 0.002 | 726 | 205 | 0.73 | 0.61, 0.88 | <0.001 |
| P for trend |  |  |  |  | <0.001 |  |  |  |  | <0.001 |  |  |  |  | <0.001 |
| **Exclude individuals with malignant cancer** |  |  |  |  |  |  |  |  |  |  |  |  |  |  |  |
| L（0-49） | 3,349 | 1,294 | — | — |  | 3,349 | 1,294 | — | — |  | 3,349 | 1,294 | — | — | — |
| M（50-79） | 12,963 | 4,183 | 0.76 | 0.70, 0.82 | <0.001 | 12,963 | 4,183 | 0.81 | 0.74, 0.87 | <0.001 | 12,963 | 4,183 | 0.80 | 0.74, 0.87 | <0.001 |
| H（80-100） | 723 | 204 | 0.62 | 0.52, 0.74 | <0.001 | 723 | 204 | 0.74 | 0.62, 0.89 | 0.001 | 723 | 204 | 0.73 | 0.61, 0.87 | <0.001 |
| P for trend |  |  |  |  | <0.001 |  |  |  |  | <0.001 |  |  |  |  | <0.001 |
| Abbreviations: CI = Confidence Interval, OR = Odds Ratio | | | | | | | | | | | | | | | |
| Model 1 : no covariates were adjusted  Model 2 : adjusted for Age and Gender.  Model 3 : adjusted for Age, Gender, Personal monthly income ≥3000 CNY, High school or above and Alcohol drinking. | | | | | | | | | | | | | | | |
